# Odontological Society of Pennsylvania

**Published:** 1893-04

**Authors:** 

**Affiliations:** Odontological Society of Pennsylvania


### ODONTOLOGICAL SOCIETY OF PENNSYLVANIA.

THE subject for discussion, "What are the best compositions for temporary fillings for a minimum period of three years?" was then considered. It was opened by

*Dr. Peirce.*—In considering the advisability of materials for temporary fillings, we must take into consideration the location of the cavity to be filled, and also the condition of the tooth. Those two things would modify my practice very much as to the selection of material. We have, of course, several preparations used for that purpose, and used very advisedly in certain teeth. If I had a cavity in the central incisor of a child, for instance, or where the patient was not able to stand a gold filling, or where there was not sufficient room to put in a gold filling, as on the mesial or distal surfaces, I should prefer to place in a filling of gutta-percha or phosphate-zinc or chloride-zinc cement, believing it would be very comfortable and thoroughly protect the tooth from decay as well as any other material, being attended with less tax upon the patient or the endurance of the child. On the other hand, if the cavity was on the masticating surface of the first molar, and I desired to insert a temporary filling, then I should prefer a zinc-phosphate filling, believing it would give more durability and would as well also protect the tooth from further progress of caries. Where the cavity is on the mesial or distal surface, whether anterior or posterior, where I desire a filling for temporary use, lasting as a minimum three years, I am in favor of using gutta-percha. With deciduous teeth I prefer the pink, because it has some advantages over the ordinary white gutta-percha. The pink gutta-percha in a child's deciduous tooth adapts itself at a low temperature to the cavity, and also has a degree of resistance that the white does not, because of its greater elasticity or the less amount of mineral substance in the material. I think these three materials—pink and white gutta-perch and zinc-phosphate—are the best. The practice must be varied according to the location of the cavities, and also by the age and endurance of the patient. I have had very good results with pink gutta-percha where I could do little more than wipe out the cavity and warm a pellet and press it in. With an irritable patient and disinclined to have anything done with the cavity, and where it is impossible to keep it dry, take out the softened part and pack in pink gutta-percha. In my hands it has produced very good results, protecting the teeth from further decay for a period certainly of three or four years.

In permanent teeth, where color is of some importance, I should use the white, but that requires more softening and does not have for the masticating surface so resisting a capacity as the pink. I meet with a great many fillings, placed in by myself and others, of zinc-phosphate, but they vary so much in different mouths that they are not reliable. I see fillings very often that have been in for three, four, or five years, and they keep their integrity well, but in other cases they have dissolved and grown imperfect at the cervical margins quite early after being put in; so that I do not consider phosphate of zinc so reliable in cavities bordering on the cervical margins as I do pink or white gutta-percha. But one of these three materials is used invariably where I want a temporary filling lasting from one to three years.

*Dr. Jefferies.*—I have pursued the plan Dr. Peirce has stated, and I find it impossible to fix upon one material. Different cases require different treatment to attain the best results, and the course he has pursued has been the same as mine, except in some where the decay is large between the teeth and it is necessary to use zinc-phosphate to make it last the longest period,—for a minimum of three years. I combine it with gutta-percha at the cervical margins, which has been the practice of a great many. In that way, as that seems to be where it is first dissolved, we get the best results. In other respects my practice is exactly the same. I use gutta-percha for front teeth and cement for back teeth, or in some cases, as on the masticating surface and where the appearance makes no difference, I use very frequently amalgam. For a temporary filling I think there is nothing better than pink gutta-percha. I often use it between children's teeth. If it wears out it is quickly and easily renewed, and it will prevent forcing of the food down between the teeth and impinging upon the gum, by reason of its filling up the entire space between the teeth.

Dr. Peirce stated that a year or so ago Dr. Brubaker tested some zinc-phosphate pellets for him, and it was found that lactic acid would destroy the zinc-phosphate very rapidly, while the pellets in the acetic acid for a year or so had suffered no visible disturbance. The doctor thought it was a fact worth putting on record that lactic acid dissolves the cement very rapidly, while acetic acid will not destroy it.

*Dr. Truman.*—This question is such a narrow one that it is difficult to talk about it. Of course there are two or three materials we can use,—gutta-percha, tin (as temporary filling), and zinc-phosphate. Those three are practically the only ones we have. I think possibly red gutta-percha is not affected by micro-organic life, while I am convinced white gutta-percha is. It decomposes on the surface with considerable rapidity. The red is, I believe, colored with vermilion; that may have a retarding influence in the development of micro-organisms. I know of no other reason, because they are practically the same. We all know that red gutta-percha will remain intact in cavities for almost an indefinite period.

I think this question of temporary fillings can hardly be considered at all without broadening the subject somewhat. It seems to me we ought to take into consideration the character of the tissue we are working upon in using temporary fillings of any kind. Their mission, of course, is to protect the teeth for a limited period. Now, how is it with teeth where the pulp is nearly exposed? Gutta-percha appears, from its expansion, to have a tendency to irritate the pulp. I think in many cases this effect is noticed, and yet we have nothing better from its lack of conductivity to thermal conditions, in children especially from twelve to fourteen years of age. I think there should be placed on record a protest against the use of gold and those high conducting materials at any age before twenty, in the majority of mouths. We always forget, I think, that the dentine of each tooth is made up of a series of tubuli, which contain organic matter capable of conducting impressions, and will place in almost anything that we may have at the moment most convenient; but we cannot do it with any degree of safety, and therefore I think we ought to avoid such temporary fillings as zinc-phosphate in extremely sensitive teeth. Oyxchloride of zinc is always more or less dangerous. I prefer to paint the walls with chloro-percha and then cover with zinc-phosphate, but I never fill temporarily on the proximal surfaces without making a compound filling. To me it is a very great error to depend upon any one material for filling in teeth of that kind. We have, as has been observed by Dr. Jack, a possibility of acid destroying the filling at the cervical border, and that fact should always be borne in mind. The red form of gutta-percha will resist the action of micro-organisms at that point better than any other. If the walls are coated with a thin layer of chloro-percha and then a layer of tin, and then upon that you build zinc-phosphate, you have very nearly a permanent filling. I am well aware from clinical experience that zinc-phosphate will dissolve in some mouths. I prefer to place in tin as a temporary filling where it is possible to use it.

*Dr. Neall.*—If I understand the question, it is, What is the best temporary filling for a minimum of three years? It strikes me that that verges on a permanent filling. I have seen many fillings supposed to be permanent that have not lasted that length of time.

I give a good deal of praise to white gutta-percha. I have used red largely, and for two years past have employed Flagg's high-heat gutta-percha, and I have been very well pleased with it, even when used on the masticating surfaces. Sometimes a filling will last not only three, but eight, years with this agent. The qualities are brought out largely by the character of fillings, as I have learned by experience. The rubber dam should be put on carefully, the cavity dried and shaped as if it were to hold a metal filling. The first piece I warm over a pipe or spirit-lamp and make it quite soft, and pack in one after another, making the first piece adhere to the cervical border, and then trim and rub down with chloroform. My experience leads me to regard Flagg's high-heat gutta-percha as the best temporary filling, verging upon a permanent filling.

Dr. Peirce stated that he attributed the value of red gutta-percha to the fact that it had vermilion in it, which was an antiseptic, and that it had a less amount of mineral substance than the white.

Dr. Jack mentioned a case where electric wires had been insulated with gutta-percha, which were placed underground, and it was found that wherever the wires passed beneath an oak-tree the insulation was destroyed. On investigation it was discovered that it was attacked by a low form of life which disintegrated it.

*Dr. Bonwill.*—I think the question was not properly framed. There are so many conditions of the teeth to be considered, and such a variety of circumstances attending the different cases, that no certain substance for filling could be named applicable to all. I favor the use of gutta-percha, and also other materials, as the different cases might require.

*Dr. Bassett.*—In my practice gutta-percha has occupied a very large place, but I have never been able to procure anything for a filling that would absolutely preserve a tooth for a minimum of three years. I am glad to repeat what Dr. Truman has said about the protection of certain parts of the cavity, preparatory to filling with zinc-phosphate. For me it has always failed very quickly at the cervical margins, so that I have come to rely on it less and less as a temporary filling, except in certain kinds of cavities. Gutta-percha will, for me, under all conditions, preserve the teeth better than any of the other so-called temporary fillings; but, of course, it has the fatal defect of wearing out wherever there is much mastication upon it, and also in proximal cavities. We are surprised often in finding fillings that we place in, supposedly for a few months, to last for many years. There are so many things involved that it seems impossible to set any rule for a certain length of time.

*Dr. Boyce.*—Dr. Neall asked the difference between gutta-perchas. It is true there is as much difference in them as there is between day and night.

The great mass of white gutta-percha is loaded with white wax or chalk, and is almost valueless. I have had pink gutta-percha become soft, as though it were rotten. So I have white gutta-percha. But, as for making it last three years, it depends upon the structure of the teeth and who is using the material.

With the great mass of mouths, especially children's, it is my sheet-anchor in preserving them from six to twenty years of age. I have a lady now in my charge with a filling on the distal surface of the second bicuspid; she masticates on it, and there is no wear. Dr. Dixon put it in twenty years ago, and yet when I place in phosphate fillings I want to see them in six months, and if they are there I am very well pleased. I cannot explain why it is different in different mouths, nor why it is called high-grade gutta-percha.

*Dr. Faught.*—I think that it is impossible to speak of any filling as being either temporary or permanent, as varying conditions make it impossible to name the time of its continuance, except, possibly, as the word is used by Dr. Ottolengui, to indicate a filling placed to cover dressings used in medication of teeth, or to tide over until convenient to fill, or some such purpose. Materials to be used can only be determined by the careful consideration of pathological conditions presented. We may discuss the mechanical and chemical properties which any material has in itself to resist disintegration, but no statement can be made as to the time these qualities will remain inherent.

*Dr. Head.*—I think it would first mean a great deal of skill to put it in, and, in the second, great judgment in choosing materials to make it last three years, unless there was absolute control of the tooth, and the formation of it when it was made. Gutta-percha is supposed to be a temporary filling, but in many cases it is far more permanent than gold. Oxyphosphate is supposed also to be a temporary filling, but in many cases it is more permanent than anything else we can use. It seems to me the thing we could best add to a filling, to make it last certainly for three years, would be brains.

The question as to the "best methods for overcoming sensibility of the dentine by local or general means" was then taken up.

Dr. Gaskill opened the subject. He stated that he had not been able to find any remedy that was reliable in all cases. Chloride of zinc he generally used. He mentioned a case where a piece of cotton caused such pain that he could not proceed. After putting in a small crystal of chloride of zinc, he was able to go ahead with comparatively no pain. The patient stated it was the most comfortable operation she had ever had performed.

Dr. Faught stated that he had used chloride of zinc with good effect. He had also used nitrate of silver with good results.

*Dr. Truman.*—To properly consider this subject, physiological conditions should be taken into consideration. It is impossible to regard the pulp as simply the remnant of the original tissue in the development of the teeth. What occasions this sensibility? All are aware there is scarcely any cavity of the mouth that micro-organisms are not in process of development, and the product being acid, constant irritation is produced, and in proportion to the density of the tooth will be the sensation. In soft-structured teeth we find increased sensibility. If we take into consideration the fact that we have not only the normal irritation of the tube contents, but also that produced by the acids spoken of, we gain a comprehension of the philosophy of the treatment of sensitive dentine. It means that we must modify the increased sensibility by using some agent that will neutralize the acidity. It was that which led me a number of years ago to recommend the use of alkalies in preference to any of the escharotics. For this purpose bicarbonate of soda is used. If this be applied properly to neutralize acidity, there will be a marked change in the sensation.

I have observed, from microscopic examination of chalky teeth, that the organic substance of the tooth is not immediately destroyed by caries, but remains with the fibrillæ in the cavity to be irritated by the increasing acidity. The fibres have not been destroyed, as in teeth of dense structure. They can be seen permeating the organic substance, which remains as a mass in the cavity, and readily raised by the excavator. With patients whose occupations bring them in contact with saccharine or acid products at confectionery-stores or vinegar-factories, I always prescribe an alkaline wash for the teeth, at least a week before commencing operations, to be used daily.

To my mind chloride of zinc is a most dangerous agent to place in a cavity. I have always dreaded its escharotic effects. Its destructive as well as its coagulating property is apparently limited only by the quantity of albumen. It is peculiar in its action. I have tested this thoroughly in minute capillary tubes. That it will be carried to the pulp sooner or later it seems to me there can be no reasonable doubt.

A large portion of the so-called obtunders have arsenic or cocaine in them. Cocaine, to be of value, should be combined with chloroform or similar agent, but its possible effects should be well considered. No one, it is supposed, would inquire whether arsenic should be placed in a tooth. There is no agent that could possibly be worse. The death of the pulp is a certain result.

The President stated that the question was asked that the consensus of opinion might be brought out against it.

*Dr. Jack.*—For ordinary sensibility I find carbolic acid, combined with oil of cloves, sufficient to alleviate, but in many instances the decalcification of the dentine produces a state of hyperæsthesia of the tubular contents which demands different treatment. No operation can proceed where the degree of sensibility is such that the slightest touch is unbearable. In these cases the local means I have found most efficient are desiccation and zinc chloride. The applicability of these agents is based on the well-known principle that if a tissue is deprived of one of its elements its normal function is impaired or destroyed.

As the use of heated air is the simplest and least painful means, I first try this. I always apply the rubber dam. The cavity is bathed with absolute alcohol, when compressed air is passed through a heated air-syringe, the bulb of which is crossed by perforated diaphragms. The air in passing becomes heated, and should be brought into contact with the tooth a little above the blood-heat. If the nozzle be held too far away the current takes with it by friction the adjacent air and is made too cool; if it is placed near, the heat is too great. The application requires to be repeated several times in some instances. Often the result is extremely satisfactory; at other times the dentine will not become dried, and there is no result.

When this is the case I do not hesitate to employ zinc chloride where the cavities are comparatively shallow, and in a long experience I have not encountered the death of the pulp from this cause. The method I follow is invariably to use rubber dam. The cavity is dried and a crystal of the salt placed in it. If the deliquescence is not immediate I add a little previously deliquesced zinc chloride and await the result. Frequently one can tie up the rubber water-tight, tuck the bag in the cheek, and go on with another case.

As the extreme sensibility of the tooth is in the part immediately beneath the soft caries, it is important to remove this, if we can, to allow the chemical to act at once where most needed, otherwise two applications may be required.

When the pain has ceased I wash out the cavity and proceed to prepare it, knowing that if the instrument reaches sensibility it has passed beyond the sphere of action of the chloride, and that any uncombined salt will have been washed out by the water and saliva.

In some cases there is not the least action by the zinc chloride, which can have but one explanation, which is that the vital force of the tissue has been sufficient to prevent the combination with its albumen. In these instances, when it is necessary to prepare the cavity and it will not receive a temporary filling of any kind, I give ether and operate at the first stage of anæsthesia whenever I can. At this stage it is more satisfactory, as the patient in this condition is able to respond to questions. I have in this stage frequently prepared the most sensitive cavities, giving a few inhalations at each recurrence of sensibility. Thus far I have experienced no indications of shock, and much less nervous depression than would follow an attempt to prepare a cavity without ether when the use of it is indicated.

Dr. Bonwill spoke of the necessity of having good sharp instruments, in order to lessen pain in operating.

The query demands an essay.

The various conditions of the dentine, involved as it is with the pulp, require antiseptic treatment.

I never use arsenic except for the destruction of the pulp. It is the best agent known, used with proper judgment.

I have tried many obtunders from electricity (to-and fro current), carrying it from the nerve-centres to the periphery or pulp, counterbalancing the pain. It will do it in many cases.

I find now that sharp excavators, burrs, and spear-pointed drills, accompanied with a very quick, full, deep inhalation, made immediately before the instrument is placed on the sensitive bone, accomplishes good results. The stroke of this should be finished before the patient fully charges the lungs. Then desist until the lungs are again inflated, thus changing; for if the cutting begins at an improper time the patient will not inhale quickly.

To produce a more profound effect, have the patient breathe one hundred to the minute, and, while yet doing this, excavate as freely as desired. The former plan generally suffices. Never remove all the carious portion at the first sitting.

Take a piece of paper charged with creosote or carbolic acid, fit it over the bottom of the cavity, and place upon this warmed gutta-percha (pink base plate), and then let it alone for weeks and months, which will not obtund further sensibility, but wedges the teeth apart by the pressure of mastication.

The operator should be very quick in his movements; all instruments should be convenient. Three things are essential to success, —*time*, gutta-percha, and creosote. Hurried excavation and filling at the same sitting will not answer.

I would never use a hypodermic injection of any kind. I have used chloroform only in moderate quantity and in special cases.

*Dr. Head.*—Many remedies satisfactory in one practitioner's hands are not so in those of another. When cocaine was first introduced, a two-per-cent., then a four-per-cent., and finally a fifty-per-cent. solution was used. The method now generally pursued is that of desiccation, in conjunction with chloride of zinc; also an alcohol spray, using it after the rubber dam is adjusted. By this treatment the operation had been performed, where before a piece of cotton applied caused the greatest pain.

Dr. Luckie thought that all the obtunders placed on the market were a vanity and vexation. There is none that will answer in all cases. Cocaine had never been very satisfactory, and he attained the best results by desiccation and by using very sharp instruments. He had used etherization with good results.

After the close of the discussion it was unanimously resolved that it is never expedient to use arsenic in any form for deadening sensitive dentine.

Adjourned.

J. Atkinson McKee, D.D.S.,
*Editor Odontological Society of Pennsylvania.*

